# Increased average number of medical publications per interviewee from 2009 to 2018: a study of 100 interviewees to an academic gastroenterology fellowship program

**DOI:** 10.1186/s12909-019-1841-2

**Published:** 2019-11-04

**Authors:** Zaid Imam, Mitchell S. Cappell

**Affiliations:** 1 Department of Internal Medicine, William Beaumont Hospital, Royal Oak, MI 48073 USA; 2 Division of Gastroenterology & Hepatology, William Beaumont Hospital at Royal Oak, MOB #602, 3535 W. Thirteen Mile Rd, Royal Oak, MI 48073 USA; 3 Oakland University William Beaumont School of Medicine, Royal Oak, MI 48073 USA

**Keywords:** Gastroenterology (GI) fellowship, Application (applicants), Medical residents, Fellowship selection (match), Medical research, Medical publications, Scholarly activity, Academic medicine, Mentorship

## Abstract

**Background:**

Gastroenterology fellowship candidates may strive to improve their qualifications for this extremely competitive fellowship.

**Objective:**

To analyze whether extreme competitiveness of gastroenterology fellowship positions has affected fellowship interview selection by statistically analyzing 13 parameters of interviewees to identify statistically significant time changes during last 10 years.

**Methods:**

Retrospective time-trend-analyses (performed 2018) on thirteen prospectively-obtained-parameters of 47 interviewees (2009–2011) vs. 53 interviewees (2016–2018) for gastroenterology fellowship. SETTING: William-Beaumont-Hospital, Royal-Oak: academic fully-accredited gastroenterology fellowship, teaching hospital of Oakland-University-William-Beaumont-School-of-Medicine, tertiary-care hospital, GI fellowship since 1973.

**Results:**

Statistically significant increases occurred from 2009 to 2011 vs. 2016–2018 in number of publications, including mean number of: abstracts (1.69 ± 0.37 vs. 7.54 ± 1.16, *p* < 0.0001); peer-reviewed articles (1.48 ± 0.30 vs. 6.13 ± 1.29, *p* < 0.0001); and total publications (3.17 ± 0.48 vs. 12.76 ± 1.99, *p* < 0.0001). Increased publications were associated with graduating from foreign medical schools (correlation coefficient = 0.26, *p* = .03), and were, surprisingly, correlated with lower letters-of-recommendation-scores (Kruskal-Wallis-statistic = 5.82, *p* = .002). USMLE-Step-1 scores significantly increased from 2009 to 2011 to 2016–2018 (235 ± 14.1 vs. 244.9 ± 13.5, *p* = 0.001) (previously reported finding). Nine other parameters did not significantly change with time.

**Conclusions:**

Current report of >four-fold-increase in publications by gastroenterology fellowship interviewees at one academic-medical-center is novel. Increased focus on scholarship by applicants may be explained by their having only three parameters to improve their credentials during residency: publications, letters-of-recommendation, and honors awarded during residency (other parameters determined before residency and immutable). Current findings may benefit medical residents/medical-residency-program-directors by focusing more on publications for applications. Association between research productivity and medical promotions likely strongly motivates medical research of residents and may motivate academic faculty. Increased exposure to research/publications may improve the clinical acumen of GI fellowship applicants by enhancing their skills in critically reading the medical literature.

## Background

Gastroenterology (GI) fellowships are extremely popular and very competitive, with only 65.9% of applicants securing a GI fellowship in the 2018 match [1]. GI fellowship interviews are an essential first step for securing a fellowship, and a greater number of interviews is highly correlated with matching into a GI fellowship program [2]. Securing an interview depends on high United States Medical Licensing Examination (USMLE) Step 1 and Step 2 (Clinical Knowledge, CK) scores, favorable recommendations, academic honors during residency (particularly chief residency), training at a reputable residency program, and research scholarship [2].

This work reports the novel finding of a quantitatively extremely large and statistically significant increase in number of research publications in peer reviewed journals by GI fellowship interviewees during the last decade, and reasons for this time trend are discussed. GI fellowship applicants, GI fellowship program directors, other GI fellowship program selection committee members, and internal medicine (IM) program directors should be cognizant of this robust trend to further promote research productivity of medical residents, and improve their chances of matching in GI fellowship positions. This finding has potentially broad implications in that more training and experience in clinical research may improve the clinical acumen of GI fellowship applicants by gaining skills in critically reading the clinical literature, and may stimulate their interest in academic medicine.

## Methods

Retrospective review conducted in 2018 of prospectively collected records of interviewees at the GI fellowship program of William Beaumont Hospital at Royal Oak (WBH-RO) from 2009 to 2018 were analyzed. The GI division sponsors a desirable, academic, GI fellowship program as evidenced by being the primary teaching hospital of a medical school (Oakland University William Beaumont School of Medicine), providing highly complex tertiary medical care (e.g. referral center for endoscopy retrograde cholangiopancreatography (ERCP) and endoscopic ultrasound), and consistently ranking among the top 25 hospitals nationally in Gastroenterology by United States (U.S.) News & World Report [3]. This GI fellowship program is approved by the Accreditation Council for Graduate Medical Education (ACGME), with no citations during the period of 2009–2018, and has been continuously in existence since 1973. The GI Division has had two fellowship positions per annum during the last 10 years. The institutional GI fellowship selection committee consists of the program director (Dr. Cappell) and three other standing members, who have remained unchanged during the last decade. The Institutional Review Board (IRB) granted IRB exemption/approval for this study on January 28, 2018 (IRB Number: 2017–382, see Statistical analysis for protocol modification by IRB).

Baseline parameters were de-identified and compiled in a database, including: individual USMLE Step 1 and Step 2 CK scores; gender; applying while chief resident vs. no chief residency; U.S. citizenship or legal permanent resident (green card holder) vs. other citizenship status (J1 or H1B visa holders); graduating from foreign vs. American medical schools; graduating from allopathic (MD) vs. osteopathic (DO) medical schools; university affiliated vs. non-university affiliated residency programs; semi-quantitative evaluation of recommendation letters; number of published articles in peer-reviewed journals; number of published abstracts; total number of scientific publications in peer-reviewed journals; and location of residency program in Midwest vs. other geographical regions, as classified by the U.S. Census Bureau geographic definitions [4].

For inclusion, publications (including original studies, review articles, case reports, case series, and editorials) had to be indexed in PubMed, which was verified prospectively by the GI fellowship selection committee chairman during the application process. Articles published in journals not indexed in PubMed or that could not be verified in PubMed at the time of the interview were excluded. Letters to the editor were counted as equivalent to abstracts. Abstracts had to be published at national (United States) meetings or conventions for inclusion; abstracts presented or published at local (hospital, city, or state) meetings or proceedings were excluded.

Recommendation letters were semi-quantitatively, graded by the program director prospectively during each application cycle, with a standard grading scale from 80 to 100, with ≤82 being a poor evaluation, 85 being an average evaluation, and ≥ 88 being a superb evaluation (for semi-quantitative grading of letters see Cappell [5]).

Comprehensive Osteopathic Medical Licensing Examination of the United States (COMLEX) step 1 and step 2 scores for osteopathic graduates were converted to equivalent USMLE Step 1 and Step 2 CK scores, respectively, according to the following published conversion formulas [6]. USMLE Step 1 score = 67.97 + (0.24) x (COMLEX Level-1 score); and USMLE Step 2 score = 102.2 + (0.18) x (COMLEX Level-2 score).

For the years 2009–2011, USMLE test scores were graded as two-digit scores by the National Board of Medical Examiners (NBME) for American medical school graduates (AMGs), and by the Educational Commission for Foreign Medical Graduates (ECFMG) for foreign medical school graduates (FMGs). The USMLE eliminated the two-digit test score in 2013, and the three-digit test scores became standard in 2013 [7]. A two-digit score of 75 is the minimal passing grade on the USMLE Step 1 and Step 2 CK examinations. The passing three-digit score has varied during the past decade. Utilizing published data, the three-digit scores corresponding to a passing grade (two-digit score ≥ 75) between the period 2006–2016 were averaged yielding an average score labelled β_1_ for USMLE Step 1 and β_2_ for USMLE Step 2, respectively [8]. The following formula was developed to convert these two-digit test scores for 2009–2011 into three-digit scores to uniformly compare three-digit test scores: coefficients were calculated by dividing β_1_ and β_2_ by 75 (the passing two-digit score) yielding coefficients of: 2.51 for USMLE Step 1, and 2.61 for USMLE Step 2, respectively, which were then multiplied by the two-digit scores to calculate three-digit scores.

All analyzed parameters were available for all interviewees, except for grades of recommendation letters which were unavailable (lost) for two interviewees in 2010 and for one interviewee in 2018, and these recommendation grades for the three applicants were necessarily excluded from analysis.

### Statistical analysis

The Institutional Review Board (IRB) modified the study protocol to require a comparison of first three vs. last three years of (instead of original protocol of comparison of first vs. last year) interviewees to better de-identify individual applicants by creating larger study populations. Comparison of three vs. three years also fortuitously increased study power by increasing the number of individuals for each of the two study populations. Individual parameters were, therefore, statistically compared for interviewees from 2009 to 2011 vs. 2016–2018.

Records of all 156 interviewees during the decade were examined, but only 100 individual interviewees in the first three or last three years were statistically compared. The total number of applicants per annum varied minimally during the decade (range of 240 to 320 per annum), with no significant time trends. The number of applicants selected for interviews per annum also varied minimally over the decade: 16.0 ± 1.0 interviewees per annum from 2009 to 2011 vs. 17.33 ± 2.31 interviewees per annum from 2016 to 2018 (*p* = 0.41).

Statistical significance of time trends for 2009–2011 versus 2016–2018 across the sample of 100 interviewees was analyzed using non-parametric statistics because the number of abstracts, articles, and publications per interviewee per annum was demonstrated to have nonparametric (non-normal) distributions by direct visual inspection of histograms, of Q-Q plots, and of box plots. Shapiro-Wilk’s test also showed a non-normal distribution (*p <* 0.0001, for normal distribution, individually for abstracts, articles, or publications). However, statistical analyses were also performed utilizing parametric comparisons with non-paired t-tests for continuous variables, and χ^2^ tests for categorical variables (which demonstrated the same or higher statistical significance for all paired analyses than nonparametric analyses).

Correlations between continuous variables or total number of publications were tested using Pearson correlation coefficients. Associations between categorical variables and total number of publications were analyzed by Kruskal-Wallis tests. *P <* 0.05 demonstrated statistical significance using two-tailed analyses. Statistical analyses were performed utilizing International Business Machines (IBM) Statistical Package for the Social Sciences (SPSS) 25, (IBM Corporation: Armonk, NY, 10504).

## Results

### Epidemiology

Interviewees were 78% male, 47% foreign medical graduates, 94.0% medical doctors (MDs), 6.0% osteopathic doctors (DOs), 86% were medical residents at (or had graduated from medical residencies that were) primary affiliates of medical schools, 36% required visas for GI fellowship, and 58% were from residency programs located in the Midwest (Table 1**)**.

**Fig. 1:**
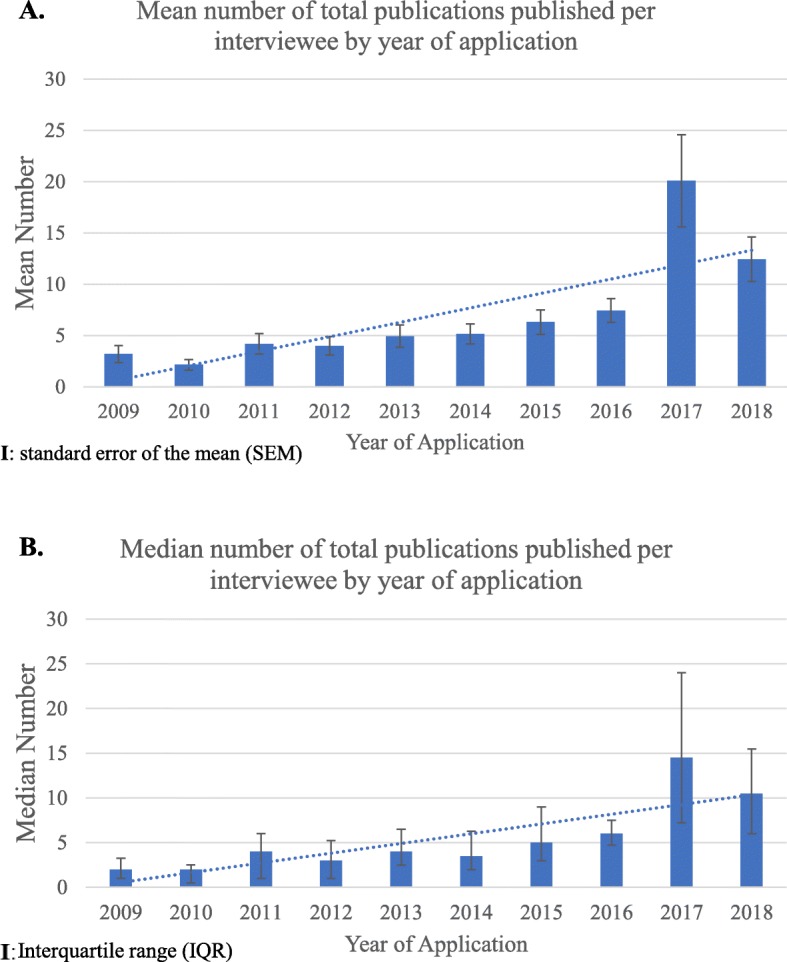
**a** Mean number of total publications (articles and abstracts) per interviewee according to year of application. Figure shows mean number and standard error of mean of total publications (including articles and abstracts) per interviewee according to year of application. Note the large increase in mean number of publications per interviewee from 2009 through 2018. Nonparametric statistical analysis of distribution of number of publications from 2008 to 2011 versus 2016–2018 showed the increase was highly statistically significant (*p <* .0001, Table 2). **b** Median number of total publications (articles and abstracts) per interviewee according to year of application. Figure shows median number and interquartile range of total publications (including articles and abstracts) per interviewee according to year of application. Note the large increase in median number of total publications per interviewee from 2009 through 2018

**Table 1:** Comparison of distribution of parameters for interviewees for GI fellowship: 2009–2011 vs. 2016–2018 using parametric statistical analysis

Categorical variables	Number (%) with parameter 2009–2011 (Total *N* = 48)	Number (%) with parameter 2016–2018 (Total *N* = 52)	*P-*value
Male (vs. Female Sex)	34 (70.8%)	44 (84.6%)	0.146
Foreign medical graduates (vs. American medical graduates)	21 (43.8%)	26 (50%)	0.554
MD graduates (vs. DO graduates)	48 (100%)	46 (88.5%)	0.027
Applied as chief resident (vs. no chief residency)	2 (4.2%)	7 (13.5%)	0.163
Medical school affiliated residency program (vs. non-affiliated residency program)	45 (93.8%)	45 (86.5%)	0.322
Residency program in Midwest (vs. elsewhere in United States)	31 (64.6%)	27 (51.9%)	0.228
U.S. Citizen or legal permanent resident (vs. foreign citizenship)	29 (60.4%)	35 (67.3%)	0.535
Mean recommendation score (evaluated semi-quantitatively, see Methods)^a^ Mean USMLE Step 1 score	86.96 ± 1.461 235.4 ± 14.1	85.1 ± 0.64 244.9 ± 13.5	<.0001* 0.001*
Mean USMLE Step 2 CK score	244.9 ± 16.7	250.8 ± 15.2	0.069*
Mean of averaged combined USMLE Step 1 & 2 scores^b^	240.1 ± 14.3	246.9 ± 13.7	0.036*
Mean number of abstracts (at national meetings)	1.69 ± 0.37	7.54 ± 1.16	< 0.0001*
Mean number of articles (listed in PubMed)	1.48 ± 0.30	6.13 ± 1.29	0.001*
Mean number of total publications	3.17 + 0.48	12.76 + 1.99	< 0.0001*

*N* Number, *MD* Medical doctor, *DO* Doctor of osteopathy, *S.D*. Standard deviation, *USMLE* United States Medical Licensing Examination, *CK* Clinical Knowledge

^a^Three individuals had missing recommendation scores (see Methods)

^b^Mean of (step-1 score + step-2 score)/2

*Utilizing independent t-tests assuming unequal variances

### Significantly varying parameters with time

SimplePara>Figure 1a illustrates time trends in *mean* number of total publications (abstracts and articles) by interviewees per annum from 2009 to 2018. Figure 1b illustrates time trends in *median* number of total publications (abstracts and articles) by interviewees per annum from 2009 to 2018. Both graphs demonstrate quantitatively large increases with time (similarly, only abstracts per interviewee per annum or only articles per interviewee per annum had large increases with time from 2009 to 2018, not illustrated)

By non-parametric statistical analyses, the 2016–2018 interviewee cohort had significantly more publications than the 2009–2011 interviewee cohort in terms of mean number of: abstracts (7.54 ± 1.16 vs. 1.69 ± 0.37, *p <* 0.0001); articles (6.13 ± 1.29 vs. 1.48 ± 0.30, *p <* 0.0001); and combined number of publications (including both abstracts and articles) (12.76 ± 1.99 vs. 3.17 ± 0.48, *p <* 0.0001)(Table 2**).** Not surprisingly, the differences were also statistically significant when analyzed using parametric statistics (Table 1).

**Table 2:** Comparison of distribution of abstracts, articles, and total publications for interviewees for GI fellowship: 2009–2011 vs. 2016–2018 using non-parametric statistical analysis

Variable	2009–2011 (*N* = 48)	2016–2018 (*N* = 52)	*P*-value^α^
Mean Number of abstracts	1.69 ± 0.37	7.54 ± 1.16	< 0.0001
Mean Number of articles	1.48 ± 0.30	6.13 ± 1.29	< 0.0001
Mean Number of publications	3.17 ± 0.48	12.76 ± 1.99	< 0.0001

^α^Statistical analysis of two tailed *p*-values computed by Mann-Whitney U test and Wilcoxon Rank Sum test

Associations between different parameters and mean number of publications, across all 100 individuals, are reported in Tables 3 and 4. Surprisingly, lower recommendation scores significantly correlated with an increased number of publications (correlation coefficient = 0.26, *p* = 0.01, Table 3). Foreign medical school graduate status was significantly associated with more publications (Kruskal Wallis test statistic = 5.82, *p* = 0.02, Table 4).

**Table 3:** Correlation coefficient (and *p*-value) between various interviewee parameters and total number of publications for GI fellowship per interviewee for 2009–2011 & 2016–2018

Parameter	Correlation Coefficient of Parameter with Number of Publications *p*-value	
USMLE Step 1 Score	.19 .06	
USMLE Step 2 CK Score	.082 .42	
Average of USMLE Steps 1 & 2 Scores	.048 .64	
Mean recommendation score (see Methods)	−.257 .01	

*USMLE* United States Medical Licensing Examination, *CK* Clinical Knowledge

**Table 4:** Association between other important parameters of interviewees and total number of interviewee publications for the analyzed years 2009–2011 and 2016–2018^a^

Individual parameters	Kruskal Wallis Statistic	*P*-value
Male Sex	2.26	.13
Foreign medical graduate	5.82	.02
Residency program in the Midwest	2.46	.12
J1 or H1B visa needed	2.58	.11
Completed chief residency year	0.17	.67
MD graduate	0.678	.41
Residency program is primary affiliate of medical school	0.04	.85

*MD* Medical doctor

^a^Analysis of correlations between individual parameters and total number of publications for 2009–2011 and 2016–2018 (not a time trend analysis)

### Invariant parameters with time

No significant differences were noted in the interviewees between 2009 and 2011 vs. 2016–2018 in percentage of males; foreign vs. American medical school graduates; graduates applying as chief resident vs. not as chief resident; USMLE Step 2 CK scores; medical school affiliated residency vs. not; geographic distribution of residency program; and U.S. citizenship or legal permanent resident status vs. other visa status (J1 or H1B visa holders) **(**Table 1**)**.

There was a non-significant time trend of more articles (4.83 vs. 2.67, *p* = 0.077), abstracts (9.17 vs. 2.17, *p* = 0.116), and total publications (14.0 vs. 4.83, *p* = 0.057) per applicant for applicants *accepted* to the GI fellowship program at the hospital from 2016 to 2018 vs. 2009–2011. This lack of statistical significance for this time trend likely resulted from under-powering because only 6 individuals accepted for GI fellowship in 2009–2011 were compared with only 6 individuals accepted for GI fellowship in 2016–2018.

## Discussion

This work identifies statistically significant time trends in parameters of GI fellowship interviewees during the past 10 years. GI fellowship applicants submit detailed applications which include standardized national test scores, residency program, recommendation letters, curriculum vitae, and personal statements. Fellowship selection committees generally offer interviews to the highest ranked candidates, based on review of these parameters. Following interviews, the institutional fellowship program selection committee ranks applicants according to these parameters, and interview evaluations. The applicants also individually rank their preferences for GI fellowship. A complex computerized mathematical algorithm then utilizes the rankings by the fellowship applicants and the fellowship selection committees to match the highest ranked applicants to the most desired available fellowship positions [9].

The last analysis by the National Residency Matching Program (NRMP) of parameters of the 2018 fellowship applicants included mean number of scholarly activities, but did not compare this data to that of prior years [2]. The previous report, published by NRMP in 2011, excluded scholarly activities [10]. The current study in addition to updating the analysis from 2011, is novel in demonstrating statistically significant and quantitatively large (greater than four-fold) increases in mean numbers of abstracts, articles, and total publications between 2009 and 2011 vs.2016–2018. This large difference suggests an increased focus on scholarly activity by GI fellowship applicants, and possibly by fellowship selection committee members for interview selection.

The currently reported finding of an increase in USMLE Step 1 scores was previously reported [7]. This trend may be partly explained by an increase in the mean scores and passing scores during the last decade [8]. In the current study, a higher number of publications was significantly associated with being a foreign medical graduate (FMG). FMGs might be concerned about reduced chances of acceptance into GI fellowships in the U.S. because of employment visa restrictions, and lack of knowledge by program committee members about the reputations of foreign medical schools from which the interviewees graduated.

A significant study limitation was analyzing only interviewees and not all applicants because only records of interviewed applicants were maintained, and the records of other applicants were destroyed to avoid the large costs of commercially storing all applicant files. Other potential study limitations include use of formulas to convert from two-digit to three-digit USMLE Step scores, and to convert from COMLEX 1 & 2 scores to USMLE Step 1 & 2 scores, respectively. Multivariate analyses encountered difficulties due to only moderate sample size and absence of a large control group.

The reported study findings are limited to a university hospital (academic medical center), located in the U.S., like the study hospital of Beaumont Hospital at Royal Oak, and may not apply to gastroenterology fellowships at regional hospitals in the U.S. because of their decreased focus on medical research. The currently reported findings may apply to other highly competitive residencies or fellowships in the U.S., such as orthopedics, but might not apply to relatively noncompetitive residencies or fellowships, such as geriatrics, in the U.S. The reported findings might not apply to gastroenterology fellowships at hospitals in other highly industrialized countries due to different medical systems.

The reported trend may arise from residents focusing on improving their credentials for this highly competitive fellowship, and their inability to improve most of their other credentials during residency, such as Step 1 and Step 2 scores, which are determined before residency. The quantitatively large increase in publications per interviewee could have alternative explanations, including increasing committee bias to interview fellows with more full-length publications, or increasing bias of applicants towards research because this hospital is the primary teaching hospital of a medical school. However, it would be difficult to ascribe the four-fold increase in publications solely to such effects. Moreover, the same four faculty have served as the members of the GI fellowship selection committee without personnel changes, and the written criteria for interviewing candidates has not changed during this ten-year-study.

The major study strength is its novel, statistically significant, findings. This work adds to the literature on postgraduate medical education. Methodological strengths include the moderately long time period of longitudinal analysis, use of prospectively collected records (that were retrospectively analyzed), analyses of multiple parameters not previously studied in NRMP reports, and insights into the increased focus of GI fellowship interviewees on scholarly activities, which likely extrapolates to the entire applicant pool.

A larger sample size might have provided a model to predict which parameters are most important to obtain interviews, and to obtain GI fellowships. This model would be useful to GI fellowship applicants, internal medicine residency program directors promoting their residents, GI fellowship program directors, other GI fellowship selection committee members, and GI researchers who seek to mentor residents.

The current findings offer significant insight into the research potential of medical residents. Medical residents who are aware of the value of research publications to advance their academic career (by achieving a fellowship in their desired medical subspecialty), may be more productive in research. While this phenomenon is reported in gastroenterology, it is also anecdotally occurring in other highly competitive fellowships such as cardiology, and in highly desirable residencies, such as orthopedics. An association between research productivity (as measured by peer-reviewed publications), and medical career advancement is likely to be a very strong motivator of medical research for trainees, and may also pertain to academic medical faculty. Academic gastroenterology faculty may use this highly significant time trend to mentor and publish more research papers in gastroenterology with medical residents interested in gastroenterology fellowship positions (as has occurred with Dr. Cappell during the last 10 years). Applicants for highly competitive fellowships (e.g. gastroenterology) or residencies (e.g. orthopedics) should become aware of the increasing standards for academic research productivity, likely fueled by intense competition for these highly desirable clinical careers.

The current statistically significant trend of greater research productivity by residents (applying for GI fellowships) could improve their clinical acumen as internists and aspiring gastroenterologists. From their research experience, residents learn about the challenges, difficulties, and limitations of research. They may also become more critical readers of the medical literature, aspire to achieve the rigorous standards of evidence-based medicine, question unsubstantiated clinical dogma, and potentially stimulate their interest in academic medical careers.

## Conclusion

A highly statistically significant time trend and more than four-fold increase in mean number of publications per interviewee for gastroenterology fellowship positions is reported from 2009 to 2011 to 2016–2018. This increase is attributed to intense competition for gastroenterology fellowship positions. This has important implications for applying residents, internal medicine program directors, and gastroenterology fellowship selection committees in terms of higher standards for research scholarship productivity, and for senior gastroenterology researchers who may want to mentor residents for research projects.

## Data Availability

Data is not shared because data is highly sensitive in that it involves trainees. It also includes data that are not considered conventional identifiers (e.g. medical school of applicants) that could with substantial detective work lead to identification of individuals.

## Abbreviations

ACGME: Accreditation Council for Graduate Medical Education
AMGs: American medical school graduates
CK: Clinical knowledge
COMLEX: Comprehensive Osteopathic Medical Licensing Examination
DO: Doctor of osteopathy
ECFMG: Educational Commission for Foreign Medical Graduates
ERCP: Endoscopic retrograde cholangiopancreatography
FMGs: Foreign medical school graduates
GI: Gastroenterology
IBM: International Business Machines
IM: Internal medicine
IQR: Interquartile range
IRB: Institutional Review Board
MD: Medical doctor (allopathic)
N: Number
NBME: National Board of Medical Examiners
NRMP: National Residency Matching Program
S.D.: Standard deviation
SEM: Standard error of the mean
SPSS: Statistical Package for the Social Sciences
U.S: United States
USMLE: United States Medical Licensing Examination
WBH-RO: William Beaumont Hospital at Royal Oak
