# Correction to: Increased average number of medical publications per interviewee from 2009 to 2018: a study of 100 interviewees to an academic gastroenterology fellowship program

**DOI:** 10.1186/s12909-019-1873-7

**Published:** 2019-11-27

**Authors:** Zaid Imam, Mitchell S. Cappell

**Affiliations:** 1 Department of Internal Medicine, William Beaumont Hospital, Royal Oak, MI 48073 USA; 2 Division of Gastroenterology & Hepatology, William Beaumont Hospital at Royal Oak, MOB #602, 3535 W. Thirteen Mile Rd., Royal Oak, MI 48073 USA; 3 Oakland University William Beaumont School of Medicine, Royal Oak, MI 48073 USA

**Correction to: BMC Med Educ (2019) 19:402**

**https://doi.org/10.1186/s12909-019-1841-2**

Following publication of the original article [1], due to miscommunication during proofing, the author notified us the bellow corrections. In this Correction, correct and incorrect versions are shown.

Page 1 Correspondence.

Incorrect: * Correspondence: Zaid.Imam@beaumont.org; Mitchell.Cappell@beaumont.edu

Correct:*Correspondence: Mitchell.Cappell@beaumont.edu

Page 2 Methods (paragraph 1).

Incorrect: for endoscopy retrograde.

Correct: for endoscopic retrograde.

Page 3 Statistical analysis (paragraph 1).

Incorrect: years of (instead.

Correct: years (instead.

Incorrect: year) interviews.

Correct: year) of interviewees.

Page 3 Results (Significantly varying parameters with time).

Incorrect: SimplePara>Fig. 1a illustrates.

Correct: Fig. 1a illustrates.

Page 4 Table 1.

Incorrect: Is missing a row in the middle of the table which should be in the table as a second header.

Correct: The corrected Table 1 is presented below.

**Table 1:** Comparison of distribution of parameters for interviewees for GI fellowship: 2009–2011 vs. 2016–2018 using parametric statistical analysis

Categorical variables	Number (%) with parameter 2009–2011 (Total *N* = 48)	Number (%) with parameter 2016–2018 (Total *N* = 52)	*P-*value
Male (vs. Female Sex)	34 (70.8%)	44 (84.6%)	0.146
Foreign medical graduates (vs. American medical graduates)	21 (43.8%)	26 (50%)	0.554
MD graduates (vs. DO graduates)	48 (100%)	46 (88.5%)	0.027
Applied as chief resident (vs. no chief residency)	2 (4.2%)	7 (13.5%)	0.163
Medical school affiliated residency program (vs. non-affiliated residency program)	45 (93.8%)	45 (86.5%)	0.322
Residency program in Midwest (vs. elsewhere in United States)	31 (64.6%)	27 (51.9%)	0.228
U.S. Citizen or legal permanent resident (vs. foreign citizenship)	29 (60.4%)	35 (67.3%)	0.535
Numerical variables	Parameter Mean ± S.D. 2009–2011 (Total *N*=48)	Parameter Mean ± S.D. 2016–2018 (Total *N* = 52)	*P-*value
Mean recommendation score (evaluated semi-quantitatively, see Methods)^a^	86.96 ± 1.461	85.1 ± 0.64	<.0001*
Mean USMLE Step 1 score	235.4 ± 14.1	244.9 ± 13.5	0.001*
Mean USMLE Step 2 CK score	244.9 ± 16.7	250.8 ± 15.2	0.069*
Mean of averaged combined USMLE Step 1 & 2 scores^b^	240.1 ± 14.3	246.9 ± 13.7	0.036*
Mean number of abstracts (at national meetings)	1.69 ± 0.37	7.54 ± 1.16	< 0.0001*
Mean number of articles (listed in PubMed)	1.48 ± 0.30	6.13 ± 1.29	0.001*
Mean number of total publications	3.17 + 0.48	12.76 + 1.99	< 0.0001*

*N* Number, *MD* Medical doctor, *DO* Doctor of osteopathy, *S.D*. Standard deviation, *USMLE* United States Medical Licensing Examination, *CK* Clinical Knowledge

^a^Three individuals had missing recommendation scores (see Methods)

^b^Mean of (step-1 score + step-2 score)/2

*Utilizing independent t-tests assuming unequal variances

Page 5 Bottom, right column

Incorrect: 2009 and 2011 vs.2016–2018.

Correct: 2009–2011 vs. 2016–2018.

Page 6 Table 3.

Incorrect: It should have 3 columns and not 1 column.

Correct: The corrected Table 3 is presented below

**Table 3:** Correlation coefficient (and *p*-value) between various interviewee parameters and total number of publications for GI fellowship per interviewee for 2009–2011 & 2016–2018

Parameter	Correlation Coefficient of Parameter with Number of Publications	*p*-value
USMLE Step 1 Score	.19	.06
USMLE Step 2 CK Score	.082	.42
Average of USMLE Steps 1 & 2 Scores	.048	.64
Mean recommendation score (see Methods)	−.257	.01

*USMLE* United States Medical Licensing Examination, *CK* Clinical Knowledge

Page 6 Under Table 3 (paragraph 4)

Incorrect: are.

Correct: were.

Incorrect: ten-year-study.

Correct: ten-year-study-period.

Page 7 Author’s contributions.

Incorrect: as mentor for Dr. ZI. Both authors read and approved the final manuscript.

Correct: as mentor for Dr. ZI. Both authors are equal and primary authors. Both authors read and approved the final manuscript.

Page 7 Competing interests.

Incorrect: Gastroenterology.

Correct: Gastrointestinal.
